# Dynamic MR of Muscle Contraction During Electrical Muscle Stimulation: Potential Application to the Evaluation of Neuromuscular Diseases

**DOI:** 10.1002/nbm.70176

**Published:** 2025-11-17

**Authors:** Francesco Santini, Michele Giovanni Croce, Xeni Deligianni, Marta Brigid Maggioni, Matteo Paoletti, Leonardo Barzaghi, Niels Bergsland, Arianna Faggioli, Giulia Manco, Chiara Bonizzoni, Ning Jin, Sabrina Ravaglia, Anna Pichiecchio

**Affiliations:** ^1^ Basel Muscle MRI, Department of Biomedical Engineering University of Basel Basel Switzerland; ^2^ Department of Radiology University Hospital Basel Basel Switzerland; ^3^ Department of Brain and Behavioural Sciences University of Pavia Pavia Italy; ^4^ Advanced Imaging and Artificial Intelligence Research Unit, Neuroradiology Department IRCCS Mondino Foundation Pavia Italy; ^5^ Department of Neurology, Jacobs School of Medicine and Biomedical Sciences, Buffalo Neuroimaging Analysis Center University of Buffalo, the State University of New York Buffalo New York USA; ^6^ Servizio di Diagnostica per Immagini ICS Maugeri spa SB, IRCCS Montescano Montescano Italy; ^7^ Cardiovascular MR R&D Siemens Medical Solutions USA, Inc. Cleveland Ohio USA; ^8^ IRCCS Mondino Foundation Pavia Italy

**Keywords:** dynamic MRI, muscle, neuromuscular diseases, neuromuscular electrical stimulation

## Abstract

Thanks to the rapid evolution of therapeutic strategies for muscular and neuromuscular diseases, the identification of quantitative biomarkers for disease identification and monitoring has become crucial. Magnetic resonance imaging (MRI) has been playing an important role by noninvasively assessing structural and functional muscular changes. This exploratory study investigated the potential of dynamic MRI during neuromuscular electrical stimulation (NMES) to detect differences between healthy controls (HCs) and patients with metabolic and myotonic myopathies. The study included 14 HCs and 10 patients with confirmed muscular diseases. All individuals were scanned with 3 T MRI with a protocol that included a multi‐echo gradient echo sequence for fat fraction quantification, multi‐echo spin‐echo for water T2 relaxation time calculation, and 3D phase contrast sequences during NMES. The strain tensor, buildup, and release rates were calculated from velocity datasets. Results showed that strain and strain buildup rate were reduced in the soleus muscle of patients compared to HCs, suggesting these parameters could serve as biomarkers of muscle dysfunction. Notably, there were no significant differences in fat fraction or water T2 measurements between patients and HCs, indicating that the observed changes reflect alterations in muscle contractile properties that are not reflected by structural changes. The findings provide preliminary evidence that dynamic muscle MRI during NMES can detect abnormalities in muscle contraction in patients with myotonia and metabolic myopathies, warranting further research with larger, more homogeneous patient cohorts.

## Graphical abstract

This exploratory study investigated dynamic MRI during neuromuscular electrical stimulation (NMES) as a biomarker for muscular diseases. Fourteen healthy controls and ten patients with metabolic and myotonic myopathies underwent 3T MRI scanning. Results showed reduced strain and strain buildup rates in patients' soleus muscles compared to controls, while fat fraction and water T2 measurements showed no significant differences. These findings suggest dynamic MRI with NMES can detect functional impairment not captured by structural imaging, offering potential for disease monitoring.

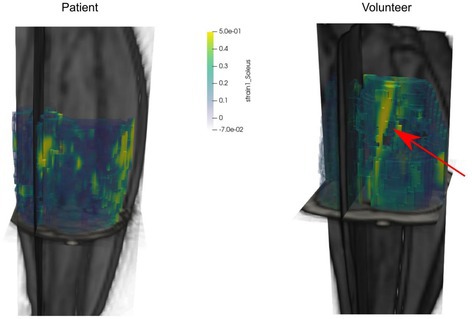

**Abbreviations**
FSHD: facioscapulohumeral dystrophy
GRE: gradient echo
HC: healthy controls
MVF: maximum voluntary force
NMD: neuromuscular disease
NMES: neuromuscular electrical stimulation
SCN4A: sodium voltage‐gated channel alpha subunit 4
TUG: timed‐up‐and‐go

## 1 Introduction

Neuromuscular diseases (NMDs) usually affect children from a young age and can be fatal. The most common causes of neuromuscular diseases are rare genetic conditions that have long been untreatable. In recent years, however, new therapies (including gene therapy) are opening new doors for affected individuals, albeit for a limited number of known diseases [1, 2, 3, 4, 5]. With the development of novel therapies, the need for reliable biomarkers that can provide objective insight into the course of the disease and detect early therapeutic effects has arisen.

In particular, magnetic resonance imaging (MRI) has been proven to provide exceptional flexibility and accuracy, with the capability of assessing muscle geometry and morphology, tissue composition, and function, over the whole organ of interest, in the same examination [6]. However, despite the importance of MRI in characterizing NMDs, it is still unable to provide a truly comprehensive picture of the muscle composition and functionality, with the most critical aspect being the quantitative assessment of interstitial muscle fibrosis.

The evaluation of muscle status in dystrophic diseases has so far mostly relied on T1‐weighted imaging for the assessment of chronic muscular changes and on fat‐suppressed T2‐weighted imaging for the assessment of the acute activity of the disease [7, 8, 9]. Although these methods are fast and able to provide a rough staging of the disease [10], they are increasingly complemented by quantitative assessments, that is, methods that can provide an objective quantification of the underlying tissue characteristics [11, 12]. Depending on the investigated pathology, two key techniques include fat‐suppressed T2 quantification methods, given their ability to accurately describe the activity of the disease, being sensitive to inflammatory edema and other processes affecting water distribution in the muscle [13, 14], and three‐dimensional fat/water quantification [15], which can potentially depict muscle morphology with great accuracy and can provide an objective measurement of how the muscle fibers have been permanently compromised. Alongside these primary techniques, MR‐based diffusion‐sensitive methods (diffusion‐weighted and diffusion‐tensor imaging) [16], and 31P spectroscopy for the evaluation of muscle metabolism [17] have shown promising traits for the characterization of the muscle, although not routinely used in clinical practice, and limitedly as clinical trial endpoints.

Although promising, however, these markers do not highlight all aspects of the pathology as they only focus on the acute and irreversible replacement stages [18]. Accurate markers of other aspects of the muscular microstructure are needed to obtain a better understanding of the clinical course of various muscle pathologies and to achieve higher levels of accuracy in the diagnosis and monitoring of disease progression.

Dynamic imaging of muscle contraction is one potential functional marker that has already been successfully applied in healthy individuals, especially in the context of aging [19, 20, 21, 22]. The derived parameters of strain and strain rate are sensitive to the age‐related physiological changes of the muscle, resulting in a reduction of these two quantities. These parameters reflect the ability of the muscle to deform when performing its natural function of contracting to generate an output force.

In order to elicit a periodic contraction of the muscle, required for high‐temporal resolution imaging by using methods such as phase contrast acquisitions, neuromuscular electrical stimulation (NMES) can be used in synchronization with the acquisition, which can provide reproducible results [23, 24]. This particular technique has been recently used in a study on patients with facioscapulohumeral dystrophy (FSHD), yielding promising results on the possibility of using the derived parameters of strain and contraction and relaxation rates as functional biomarkers of disease [25]. This study was however limited to a single slice acquisition of the thigh.

Due to preliminary evidence showing a reduction in strain and strain rate along with a decrease in functionality of the muscle, it can be hypothesized that these markers can also be reduced in neuromuscular diseases affecting muscle functionality. We analyzed muscular and neuromuscular diseases likely associated with changes in contraction parameters (stiffness, relaxation time), such as myotonias (dystrophic and nondystrophic), as well as those in which repeated exercise is expected to cause transient weakness, such as metabolic myopathies. Thus, in this study, we are expanding the investigation to a wider spectrum of diseases affecting the skeletal muscle with an exploratory analysis of 10 patients with dystrophic and nondystrophic myotonias and metabolic myopathies, and 14 healthy controls (HCs), focusing on time‐resolved, three‐dimensional acquisition of the triceps surae during electrical muscle stimulation.

## 2 Experimental

### 2.1 Study Population

Fourteen HCs (5 male, median age 50 years, range: 35–62) and 10 patients (6 male, median age 46 years range: 25–66) were prospectively recruited for this study. The patients had a confirmed diagnosis of muscular or neuromuscular disease, and specifically: myotonic dystrophy type II and type I, chloride channel myotonia, SCN4A channelopathy, and McArdle disease. The reason for the selection of patients with McArdle disease rather than metabolic myopathies was based on the fact that this disease is characterized by transitory weakness induced by short‐time exercise/muscle contraction, a condition that may give the opportunity to appreciate, by dynamic MRI, whether a failure in muscle ability to contract in response to muscle electrical stimulation can be detected. Moreover, McArdle disease is not associated with structural muscle degeneration at the leg, so that we can eventually appreciate a failure of the muscle to contract, due to an insufficient production of ATP related to myophosphorylase deficiency, and thus not influenced by structural abnormalities. Among myotonias, we selected three patients with myotonic dystrophy in the early disease stage (without calf muscle wasting) and five with nondystrophic myotonias caused by chloride channel mutations (three patients) or sodium channel mutations (two patients). Details on patients' demographics, clinical features, and diagnosis are in Table S1. Only one patient was taking medications for myotonia at the time of MRI. Because the MRI analysis was performed on lower limbs, the severity of myotonia as well as the functionality of lower limbs was assessed by the timed‐up‐and‐go test (TUG), in which the patients were instructed to stand up, walk 6 m and sit back down again.

All subjects gave their written informed consent to participate. The study conformed to the Declaration of Helsinki, and the experimental procedures were approved by the local ethics committee.

### 2.2 MR Image Acquisition and Processing

During a preparation phase, two sets of gel‐based electrodes were placed on the subject's right leg. The stimulation intensity was tested, and the evoked force was measured before the scan with a custom‐made MR‐compatible sensor [26], as well as the maximum voluntary force (MVF) in the same position. The stimulation current was established immediately before the scan as the current required to elicit a force of approximately 10% of the MVF, to ensure patient compliance with the discomfort of the stimulation and to avoid fatigue during the exercise, which would result in the output force changing over time [24].

For the stimulation, a commercial two‐channel NMES device (EM49, Beurer GmbH, Ulm, Germany) was used to induce periodic muscle contraction of the calf muscles. Biphasic stimulation with rectangular pulses was applied (pulse width, 400 μs; pulse frequency, 80 Hz; contraction duration, 750 ms; release duration, 750 ms). The foot was locked at a 90° angle, thus obtaining an isometric contraction paradigm.

During the MR acquisition, the second channel of the stimulator was converted to a trigger signal for the scanner by an in‐house developed electronic device [23] for synchronization of the dynamic MR acquisition with the stimulation.

The stimulation and trigger‐generation equipment was kept outside the scanner room and the electrical stimuli and the trigger signal were brought to the scanner through a low‐pass radiofrequency filter installed in the waveguide [27].

The subjects were scanned on a 3‐T clinical MRI scanner (MAGNETOM Skyra, Siemens Healthineers, Forchheim, Germany). The scanning protocol included a multi‐echo gradient echo sequence for fat fraction quantification (axial 6‐point Dixon gradient echo (GRE) sequence (matrix size = 432 × 396; TR = 35 ms; TE = 1.7–9.2 ms; resolution = 1.0 × 1.0 × 5 mm3; scan time = 15 min), a multi‐echo spin‐echo sequence for the quantification of the T2 relaxation time of the water component (water T2 (wT2)—TE/TR = [10.9–185.3] ms/4100.0 ms, 17 echo times; resolution = 1.2 × 1.2 × 10.0 mm3; slice gap = 30 mm; scan time = 5 min) [14], and, lastly, a three‐dimensional phase contrast sequence for the evaluation of the contraction during NMES. In order to avoid any potential effect of muscle exercise on wT2, the preparation and the protocol timing ensured that the subject would be lying down for at least 20 min before the multi‐echo spin‐echo acquisition.

For the dynamic acquisition, a prospectively gated, highly accelerated Cartesian 4D flow research sequence using L1‐regularized wavelet‐based compressed sensing [28, 29] was placed in a sagittal orientation to cover the whole calf. The imaging protocol had the following parameters: TE/TR 4.1/8.7 ms, flip angle 10°, bandwidth 910 Hz/px, matrix size 128 × 54 × 48, resolution 2.3 × 2.3 × 2.5 mm^3^, Venc 15 cm/s, 2 k‐space lines per segment, acceleration factor 7.6. The acquisition time was approximately 5 min per dataset.

Fat fraction maps were calculated using the publicly available FattyRiot algorithm [30].

wT2 maps were calculated through a fitting of a dual‐compartment signal model simulated with the extended‐phase‐graph method. In this method, an MR signal dictionary of the multi‐echo spin‐echo acquisition was simulated for many combinations of fat fractions and water T2 values, assuming a fixed value for water and fat T1 (1400 and 365 ms, respectively) and a fat T2 automatically estimated from the subcutaneous fat of each patient. The simulation took into account the flip angle distribution across the excited slice arising from the pulse shape. The time evolution across the echoes of each voxel was then compared to each entry in the dictionary to select the combination of wT2 and fat fraction that resulted in the highest similarity with the measured signal [13].

The velocity datasets were subsequently postprocessed offline to calculate the strain tensor at each spatial location throughout the stimulation cycle [23].

The postprocessing included the calculation of the voxelwise displacement field from the three‐directional velocity field through forward/backward temporal integration. The spatial gradient of the displacement field was then calculated by applying a three‐dimensional Savitzky–Golay derivative filter, which estimates the spatial gradient while minimizing noise amplification. From the spatial gradient, through simple algebraic manipulation, the strain tensor was obtained, which represents the stretching and compression, both in direction and amount, of each voxel of the dataset, considered as an infinitesimal deformable cube subject to the contraction forces. A Python implementation of the strain calculation algorithm from phase contrast images is available at https://github.com/BAMMri/3D‐Dynamic‐Velocity.

The largest positive eigenvalue of the strain tensor, corresponding to infinitesimal lengthening of the voxel along one direction, was considered for further quantitative processing, in line with Deligianni et al. [24, 25]. The “buildup” and “release” rates, related to the speed at which the maximum contraction was reached, or the speed of the return to the relaxed state, respectively, were calculated through a sigmoid fit of the eigenvalue time curve, as described in Deligianni et al. [22].

### 2.3 Data Analysis

The datasets were segmented using the computer‐assisted segmentation software Dafne [31] and the average values over the soleus and the lateral and medial gastrocnemii were extracted for the following variables: first positive eigenvalue of the strain tensor, buildup rate, release rate, fat fraction, and water T2. The dynamic parameters were averaged over the volume of the muscle between the centers of the two NMES electrodes, as identifiable in the MR image.

Data analysis was performed in R [32]. Due to the nonnormality of the distributions, the results are presented as median values and first and third quartiles for each considered value. For each variable, the results of a standardized logistic regression modeling with the binarized subject group (HC or patient, where the “patient” was assigned a value of 1) as the dependent variable, and the normalized (mean‐detrended and divided by the standard deviation) considered variable as the independent variable are presented. The outcome of the modeling is shown in terms of odds ratios and their 95% confidence interval.

Association between the dynamic‐derived quantities (strain, buildup, and release rates) and the quantitative parameters (fat fraction and water T2) were investigated through Spearman's correlation.

The analysis script and the extracted tabular data used are available in Santini [33].

## 3 Results

TUG test was normal in but one patient with PMC (Table S1), whereas 6MWT was within normal ranges for age, sex, and bodyweight in all patients, indicating substantially mild phenotypes through all the included muscle diseases.

**FIGURE 1:**
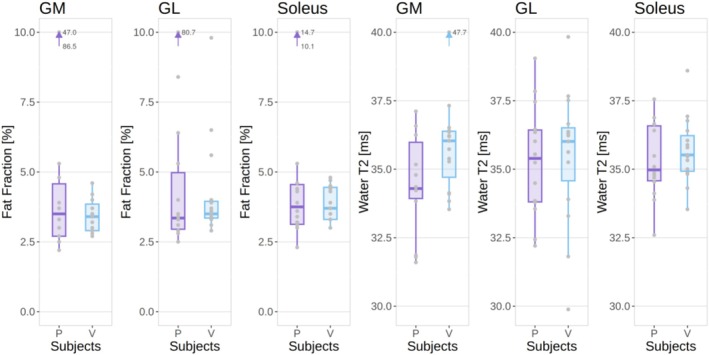
Fat fraction (left) and water T2 (right) values for each ROI in patients (P) and healthy volunteers (V). Outliers outside the limits of the plot are represented by an arrow and corresponding values.

**FIGURE 2:**
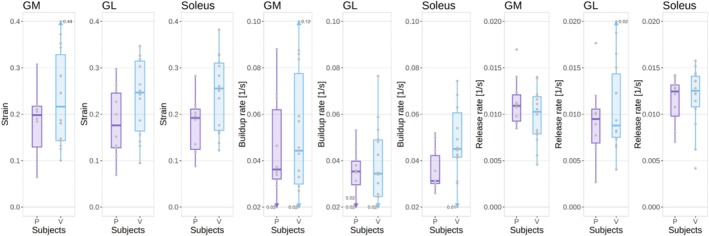
Strain (left), buildup rate (center), and release rate (right) for each ROI in patients (P) and healthy volunteers (V). Outliers outside the limits of the plot are represented by an arrow and corresponding values.

Both HCs and patients had similar median levels of fat fraction in the three muscles between 3% and 4%, although three different patients had at least one muscle with a fat fraction above 10% (Figure 1a). Similarly, median water T2 values were between 34.3 and 36.0 for all muscle ROIs in both groups (Figure 1b). The median strain, however, was higher in HCs in all three muscles, with an increase of up to 36% (0.26 vs. 0.19) in median value in the soleus of healthy volunteers with respect to patients, and 39% (but with higher variability) in the lateral gastrocnemius (Figure 2a). The buildup rate was slightly faster in the soleus (0.05 vs. 0.03 s^−1^) but not in the other muscles (Figure 2b and 2c). The values are summarized in Table 1.

**TABLE 1:** Values of the considered variables for the three muscles of interest for healthy controls and patients.

ROI	Variable	Healthy controls (median and interquartile range)	Patients (median and interquartile range)	Odds ratio (estimate and 95% confidence intervals)
	MVF (N)	80 (36–100)	78 (57–89)	0.83 (0.36–1.77)
Soleus	Fat fraction (%)	3.7 (3.3–4.5)	3.8 (3.1–4.6)	1.83 (0.77–10.01)
	Water T2 (ms)	35.5 (35.0–36.2)	35.0 (34.6–36.6)	0.73 (0.32–1.54)
	Strain	0.26 (0.17–0.31)	0.19 (0.12–0.21)	0.34 (0.10–0.89)
	Buildup rate (s^−1^)	0.045 (0.041–0.061)	0.031 (0.030–0.042)	0.38 (0.11–0.99)
	Release rate (s^−1^)	0.013 (0.011–0.014)	0.012 (0.010–0.013)	0.88 (0.37–2.08)
Medial gastrocnemius	Fat fraction (%)	3.4 (2.9–3.9)	3.5 (2.7–4.6)	4.96 (0.81 ‐ NA)
	Water T2 (ms)	36.0 (34.7–36.4)	34.3 (33.9–36.0)	0.29 (0.05–0.96)
	Strain	0.22 (0.14–0.33)	0.20 (0.13–0.22)	0.54 (0.19–1.30)
	Buildup rate (s^−1^)	0.044 (0.030–0.078)	0.036 (0.032–0.062)	0.73 (0.27–1.69)
	Release rate (s^−1^)	0.010 (0.008–0.012)	0.011 (0.009–0.012)	1.81 (0.76–5.21)
Lateral gastrocnemius	Fat fraction (%)	3.5 (3.4–4.0)	3.4 (3.0–5.0)	1.92 (0.70 ‐ NA)
	Water T2 (ms)	36.0 (34.6–36.5)	35.4 (33.9–36.4)	0.93 (0.45–1.98)
	Strain	0.25 (0.16–0.31)	0.18 (0.13–0.25)	0.47 (0.17–1.13)
	Buildup rate (s^−1^)	0.034 (0.025–0.049)	0.035 (0.030–0.040)	0.76 (0.29–1.76)
	Release rate (s^−1^)	0.009 (0.008–0.014)	0.009 (0.007–0.011)	0.67 (0.24–1.56)

*Note:* The last column represents the odds ratios of the logistic regression model applied to the variable. NA represents a value that could not be computed by the model. MVF is the Maximum Voluntary Force as measured by the dynamometer prior to the scan.

**FIGURE 3:**
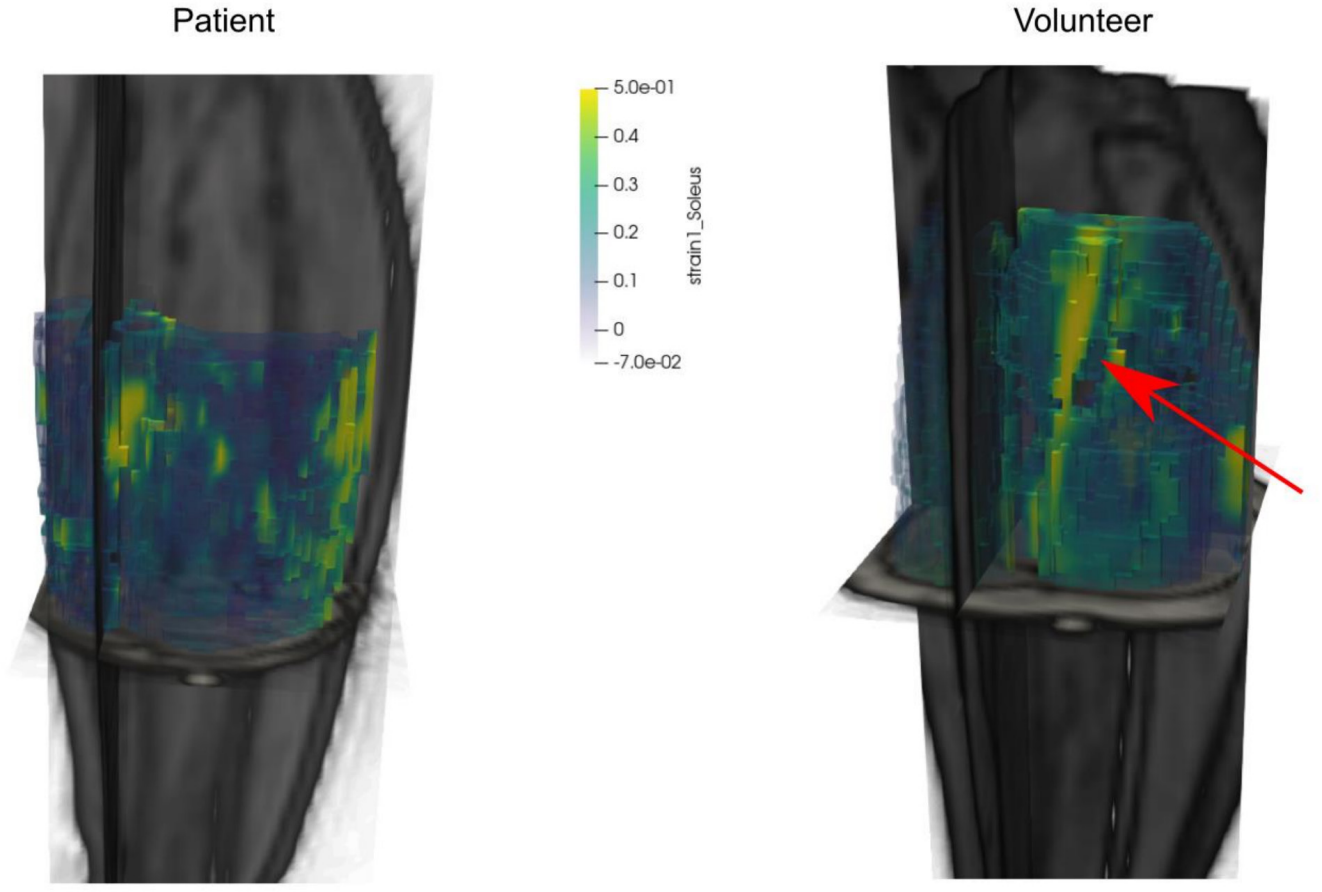
Three‐dimensional visualization of the map of the first strain eigenvector in the soleus muscle of a patient (left) and a healthy volunteer (right). A clear area of activation can be seen (marked by the red arrow) in the volunteer dataset, whereas the strain in the patient is generally lower and less localized.

An exemplary visualization of the first strain eigenvalue in the soleus of a volunteer and a patient is shown in Figure 3.

**FIGURE 4:**
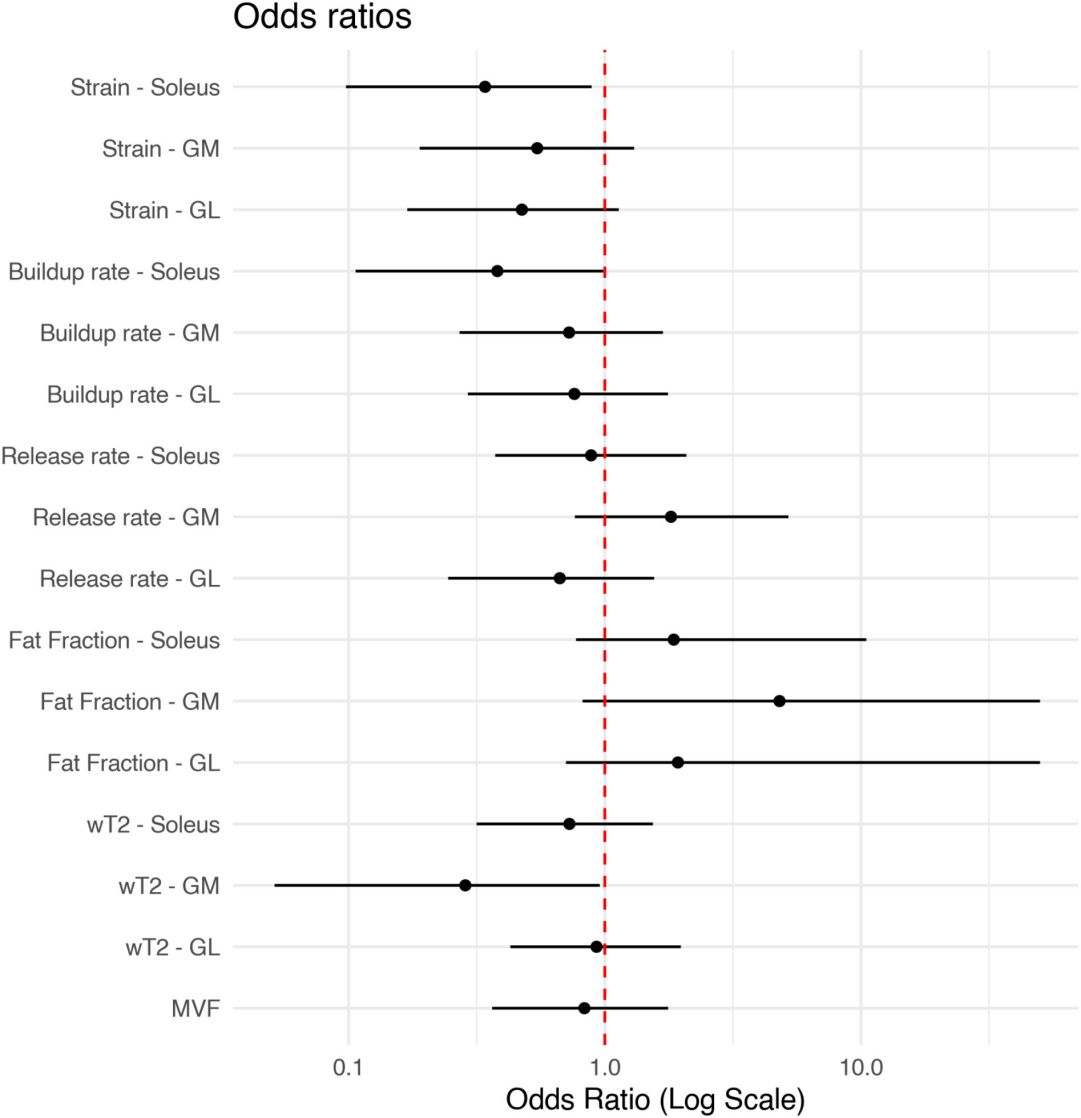
Odds ratios of the considered variables across the three ROIs with 95% confidence interval. An odds ratio of 1 means that an increase in the considered variable has no influence on the likelihood that the subject belongs to the “patient” or “healthy control” group. A higher odds ratio means that an increase in the variable increases the likelihood for the subject to be a patient, and vice versa for odds ratios lower than 1.

The logistic regression model showed a fitted odds ratio of 0.34 with a 95% confidence interval of 0.10 to 0.89 for the strain value of the soleus, meaning that an increase of one standard deviation in the strain decreases the likelihood of a subject being in the “patients” group by a factor of 0.34. Similarly, the buildup rate in the soleus has a fitted odds ratio of 0.38 with a 95% confidence interval of 0.11 to 0.99. Although with lower confidence, and with a 95% confidence interval that crosses the identity line, increased fat fraction is also a compatible indicator of belonging to the patient group (Figure 4). The logistic regression for water T2 of the gastrocnemius medialis had an odds ratio of 0.29 (confidence interval 0.05–0.96), driven by a single outlier in the volunteer group with a non–physiologically high T2 value, possibly due to physical activity before the scan or image artifact.

The MVF did not appreciably differentiate between volunteers and patients with an odds ratio of 0.89 (confidence interval 0.36–1.77).

**FIGURE 5:**
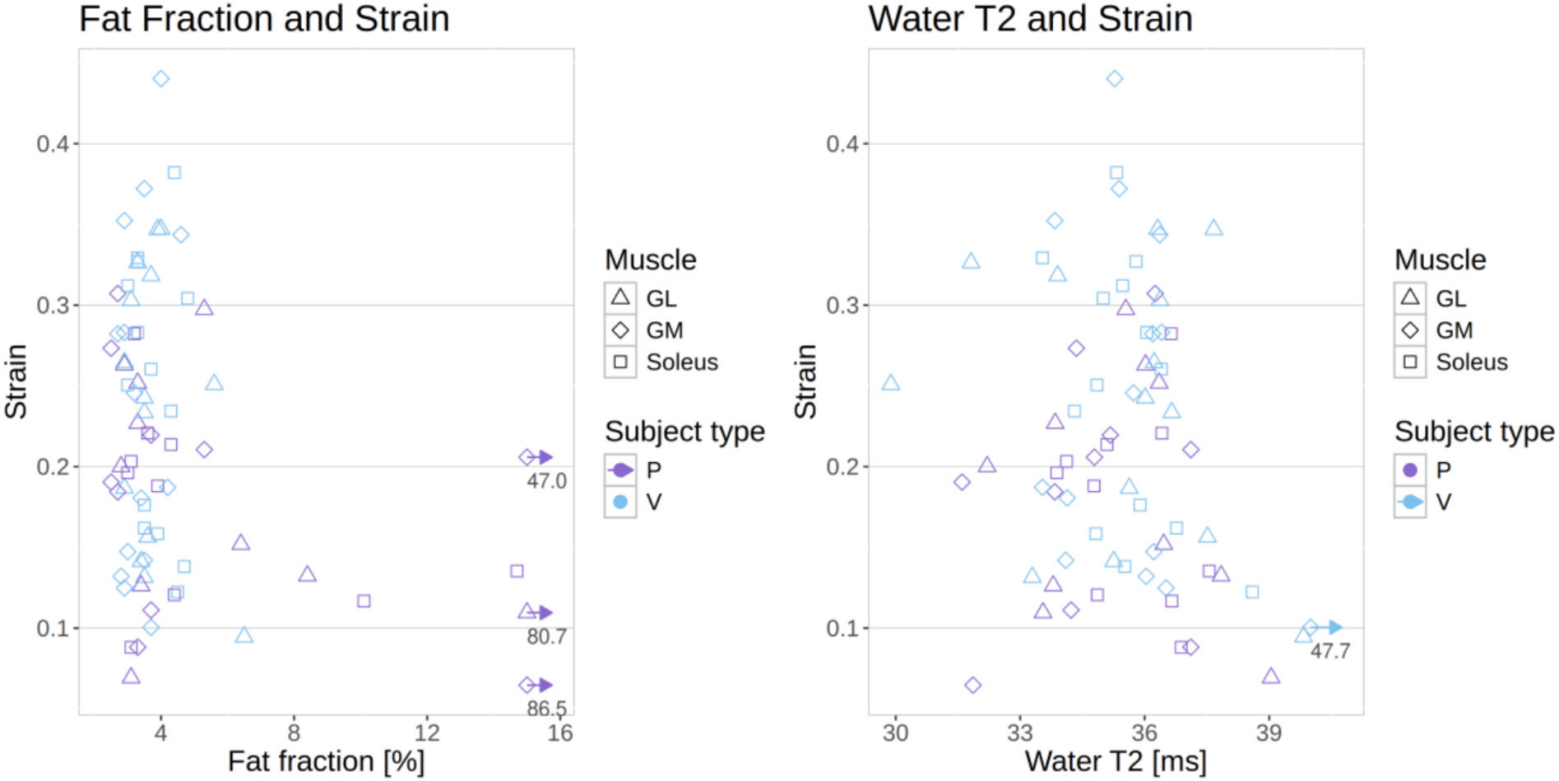
Plots of the strain vs. the fat fraction (left) and water T2 (right), for all subject types and muscle ROIs. The correlation between the variables is negligible. Outliers outside the limits of the plot are represented by an arrow and corresponding values.

Correlations between the quantitative and the dynamic variables were negligible, with the highest correlation observed being of −0.188 between strain and fat fraction (Table 2; Figure 5).

**TABLE 2:** Spearman's correlation coefficients between the dynamic and the quantitative variables.

	Fat fraction	Water T2
Strain	−0.188	−0.147
Buildup rate	0.103	−0.033
Release rate	0.033	−0.112

## 4 Discussion

In this exploratory study, we investigated the potential of dynamic MRI during NMES to detect differences between HCs and patients affected by metabolic and myotonic myopathies. Previous studies have shown changes in dynamic MRI parameters with age, possibly due to muscle fiber degeneration/loss and other structural abnormalities [20, 21, 22]. Because most neuromuscular diseases are associated with muscle fiber loss and fat replacement, and because these structural abnormalities are expected to influence the evaluation of dynamic MRI parameters, we decided to focus on diseases such as myotonias and metabolic myopathies, which are usually not associated with fixed weakness and muscle degeneration, at least in the early stages.

Myotonia is characterized, clinically and on neurophysiologic testing, by muscle hyperexcitability and delayed relaxation leading to stiffness or transient weakness, induced by voluntary movement, percussion, changes in temperature, or electrical stimulation. In chloride and sodium channel myotonia, as well as in dystrophic myotonia, the stiffness is prominent with the first movements following a period of rest, and then diminishes and may even disappear. On the contrary, in paramyotonia, stiffness increases with repeated exercise. Beyond stiffness, myotonia may be associated with transient weakness, especially with repeated exercise and especially in the paramyotonia phenotype of sodium channel myotonia. The increase in Na+ currents leading to stiffness or paralysis in sodium channelopathies is more pronounced in type II vs. type I muscle fibers.

The key finding is that strain and strain buildup rate were reduced in the soleus muscle of patients compared to HCs, suggesting that these parameters could serve as biomarkers of muscle dysfunction in muscular and neuromuscular diseases. Although other studies detected structural MRI changes in patients with nondystrophic myotonias [34, 35], we did not observe any changes in muscle structure, as assessed by FF/water T2, possibly in view of younger age or milder phenotype, with all but one patient performing well on the TUG test. On the contrary, we did not detect changes in FF in patients with myotonia, possibly in view of a milder clinical phenotype. Thus, the lack of significant differences both in conventional imaging and in FF and water T2 measurements between patients and HCs in our study supports the notion that the observed changes truly reflect a change in muscle contractile properties, independently of changes in muscle structure/composition detectable by currently established methods.

This finding is in line with existing literature on the subject of dynamic muscle imaging. Although no comparable study on patients with muscular or neuromuscular diseases has been conducted so far, results from studies in an aging population [19, 21, 22], and induced atrophy [36] show similar trends in the investigated markers. What microstructural or physiopathological changes can lead to this finding is quite speculative. One potential explanation resides in the increase in stiffness of the muscular extracellular matrix due to increased collagen deposition and cross‐linking, which occurs naturally with age [37] and is also characteristic of some neuromuscular diseases, such as Duchenne muscular dystrophy [38].

The previous study on facioscapulohumeral dystrophy [25] did not show the trend that we could find here when comparing HCs with patients; however, the previous study had the serious limitation of being limited to a single acquired dynamic slice, which reduced its comprehensiveness in evaluating the muscle, and introduced the additional uncertainty of the slice placement in the measurement.

Although all three muscles of the triceps surae show a similar trend, the difference between patients and HCs is more pronounced in the soleus muscle. This can be explained by the placement of the distal electrode just below the belly of the gastrocnemii, thus favoring soleus activation.

As an alternative to NMES, measurement of muscle motion during voluntary contractions could have been used. Voluntary contractions can achieve a higher overall elicited force, and a more physiological activation of the target muscles [39]. However, they require a different setup than the one available for this study, with a force feedback to the subject during the acquisition, and the necessity for patient compliance and ability to follow a periodic contraction pattern. This has been proven difficult in a previous study on a population of individuals with cerebral palsy [40].

This work has several limitations. Most importantly, the group of patients is rather small and heterogeneous. This is only in part dictated by the availability of the patient pool in the institution where the scans took place: indeed, our aim was to investigate a group of muscle disorders that, through different mechanisms, show an abnormal muscle contraction in response to electrical stimulation, and to explore whether any of these exhibit a recognizable pattern of parameters obtained through dynamic MRI. Based on our results, further studies could be conducted on more homogeneous groups of patients with nondystrophic myotonias and metabolic myopathies. Focusing on larger patient groups with the same pathology will help better define the applicability of dynamic MRI for the evaluation of neuromuscular diseases. Moreover, some of the chosen phenotypes (e.g., myotonia and paramyotonia) are expected to behave in an opposite way after voluntary contraction or repetitive electrical stimulation, with paramyotonia worsening and myotonia improving with repetition. Thus, the assessment of buildup, strain, and release within the same time frame may nullify the differences, if any, meaning that if the changes in contractility are not synchronous, and if the time frame is too long, an average is observed. Another potential source of variability is that the selected disorders could be that myotonias may be associated with increased muscle bulk secondary to hypertrophy from the involuntary exercise caused by myotonic contractions; we do not know the impact of this increased bulk on strength and quality of contraction.

Another limitation was that for this protocol, acquisition of the elicited force was only available before the scan, but not during the scan. So, although the standardization of the force, which is currently the most repeatable method for this kind of acquisition [24], was done outside the scanner room, it could not be repeated at the exact moment of scanning. This possibly introduced an additional factor of intersubject variability in the measurements.

As remarked previously [25], the negligible correlations found between strain, fat fraction, and water T2 suggest that these parameters provide complementary information on muscle status. Although fat fraction reflects the degree of muscle–fat replacement and water T2 in the absence of exercise is sensitive to edema, inflammation, and, generally, water distribution in muscular tissue [41], strain and strain rate are markers of the active contraction process. Combining these different imaging biomarkers could provide a more comprehensive evaluation of muscle health in muscular and neuromuscular diseases.

In conclusion, this study provides preliminary evidence that dynamic muscle MRI during NMES can detect abnormalities in muscle contraction in patients with myotonia and metabolic myopathies, independent of structural MRI abnormalities. The strain and buildup rate in the soleus muscle appear particularly promising as potential biomarkers. However, further research is needed to confirm these findings in larger and more homogeneous patient cohorts, optimize the acquisition protocol, and elucidate the relationship between dynamic MRI parameters and clinical outcomes. By combining dynamic and quantitative MRI techniques, a multiparametric imaging approach could greatly enhance our understanding and management of neuromuscular disorders.

## Author Contributions

**Francesco Santini:** conceptualization, methodology, software, formal analysis, visualization, writing–original draft, funding acquisition. **Michele Giovanni Croce:** investigation, data curation, writing–original draft. **Xeni Deligianni:** methodology, software, writing–review and editing. **Marta Brigid Maggioni:** software. **Matteo Paoletti:** investigation, methodology, data curation. **Leonardo Barzaghi:** methodology, data curation. **Niels Bergsland:** methodology, writing–review and editing. **Arianna Faggioli:** investigation. **Giulia Manco:** investigation. **Chiara Bonizzoni:** investigation. **Ning Jin:** software, resources. **Sabrina Ravaglia:** conceptualization, methodology, investigation, supervision, writing–original draft. **Anna Pichiecchio:** conceptualization, methodology, investigation, supervision, project administration, funding acquisition, writing–original draft.

## Conflicts of Interest

Ning Jin is an employee of Siemens Healthineers.

No other author has any conflict of interest to report related to the subject of this manuscript.

## Supporting information


**Table S1** Demographic and disease features of the patients and clinical phenotype.

## Data Availability

The data and the code used to generate the figures of this article are openly available in Zenodo at https://zenodo.org/records/13169701. Raw imaging data are available upon justified request addressed to anna.pichiecchio@mondino.it.
