# Supplementary material for: Dynamic MR of Muscle Contraction During Electrical Muscle Stimulation: Potential Application to the Evaluation of Neuromuscular Diseases
Source: NMR Biomed. 2025 Nov 17;38(12):e70176. doi: 10.1002/nbm.70176 (PMC12623070; doi:10.1002/nbm.70176)
Supplement: Supplementary file 1 — Table S1 Demographic and disease features of the patients and clinical phenotype. [file NBM-38-e70176-s001.docx]

Supplementary Table 1 - Demographic and disease features of the patients and clinical phenotype

| Patient_number | Sex | Age | Gene | Mutation | Phenotype (?) | Timed Up and Go | MRI FF/water T2 | myotonia treatment |
| --- | --- | --- | --- | --- | --- | --- | --- | --- |
| P01-2 | M | 66 | ZFN9 | CCTG repeat Intron 1 > 75 | dystrophic myotonia type 2 | N | FF |  |
| P02-1 | M | 54 | CLCN1 | c.2680C>T (p.R894X) | dominant MC | N | - |  |
| P03-1 | M | 25 | CLCN1 | c.2680C>T (p.R894X) | dominant MC | N | - |  |
| P04-1 | F | 54 | SCN4A | Val1589Met | sodium channel myotonia | N | - |  |
| P05-1 | M | 46 | CLCN1 | c.180+3A>T intron 1; 1182_1186delTGGAA, exon 11 | recessive MC | N | - | mexiletine |
| P08-2 | F | 46 | ZFN9 | CCTG repeat Intron 1 > 75 | dystrophic myotonia type 2 | N | - |  |
| P10-1 | M | 56 | SCN4A | P1313M | PMC | P | FF |  |
| P11-2 | F | 68 | DMPK | E1 | dystrophic myotonia type 1 | N | FF |  |
| P12-3 | F | 46 | PYGM | R50X / IVS20-1 | McArdle | N | - |  |
| P14-3 | M | 29 | PYGM | c.148C>T (p-Arg50X) and c.2262delA (p.Lys754Asnfs* | McArdle | N | - |  |

Demographic and disease features of the patients and genetic diagnosis. The functional impact of myotonia /myopathy was assessed at the lower limbs only, by the Timed -Up-and-Go test, that was divided as Normal vs Pathologic
